# Replicating motivational and cognitive-motivational typologies of doping attitudes among recreational fitness athletes: implications for harm reduction and risk communication

**DOI:** 10.1186/s12954-026-01546-3

**Published:** 2026-09-23

**Authors:** René Paasch, Gunnar Mau

**Affiliations:** 1 Department of Psychology, German University of Health and Sport, Berlin, Germany; 2 Department of Marketing and Retailing, Magdeburg-Stendal University of Applied Sciences, Breitscheidstr. 2, 39114 Magdeburg, Germany

## Abstract

**Background:**

The non-medical use of image- and performance-enhancing drugs (IPEDs) in recreational fitness represents an important public health and harm-reduction challenge. While prevalence estimates provide useful benchmarks, they often obscure the motivational and cognitive-motivational heterogeneity underlying substance-use decisions and prevention needs in gym populations.

**Objective:**

This study re-examined a previously published typology and evaluated its reproducibility of doping attitudes and prevention needs among German recreational fitness athletes using selected constructs from the Theory of Planned Behavior (TPB) and the Health Belief Model (HBM), complemented by qualitative illustration.

**Methods:**

A cross-sectional online survey was conducted with 624 recreational athletes (64.1% male, 34.5% female, 1.4% diverse; Mage = 37.1, SD = 10.0). After data cleaning, the analytical sample comprised 550 participants. Four TPB/HBM-related scales with acceptable internal consistency (α = .78–.86) served as clustering variables. Ward’s method followed by k-means clustering (k = 7, seed = 42) informed the retention of a seven-cluster solution (overall silhouette = .22; bootstrap-based reproducibility with B = 200 indicated moderate stability). Associations with lifetime use of anabolic–androgenic steroids (AAS), testosterone replacement therapy (TRT), and growth hormone (GH) were examined descriptively. Fifty-two open-ended responses to illustrate cluster-specific motivational and cognitive-motivational patterns.

**Results:**

Seven distinct motivational and cognitive-motivational profiles were identified, with descriptively distinct profile patterns across TPB/HBM-related dimensions. Small but statistically significant associations were observed for AAS and TRT, whereas GH use did not differ significantly across profiles. Qualitative accounts illustrated contrasts between value-based abstainers, ambivalent risk-takers, performance-oriented rationalisers, and low-engagement groups.

**Conclusions:**

The replication provides preliminary support for a seven-profile typology of IPED-related orientations in recreational fitness, indicating heterogeneous motivational patterns and prevention needs rather than a single risk continuum. Given the moderate stability observed in the bootstrap analyses, the typology should be interpreted cautiously at the individual level but may provide an exploratory framework for harm-reduction-oriented communication and prevention approaches in gym populations.

**Supplementary Information:**

The online version contains supplementary material available at https://doi.org/10.1186/s12954-026-01546-3.

## Introduction

The nonmedical use of image- and performance-enhancing drugs (IPEDs) in recreational fitness represents an important public health and harm-reduction challenge. In Germany, fitness centers represent one of the most common contexts for sport participation, and anabolic–androgenic steroids (AAS) and related substances have long extended beyond elite sport into recreational fitness environments [21, 39, 40]. Meta-analytic evidence estimates lifetime AAS use at approximately 3.3% in the general population, with substantially higher prevalence rates among gym populations [39]. Anabolic–androgenic steroids are well documented to enhance muscle mass and strength development and are frequently used to accelerate muscular development and appearance-related goals. At the same time, IPED use is associated with a range of cosmetic (e.g., acne, gynecomastia), systemic (e.g., cardiovascular strain, hepatic dysfunction, endocrine disruption), and psychological consequences (e.g., mood disturbances, aggression, dependency) [21, 34]. Some harms are reversible, whereas others may require long-term medical treatment or result in permanent impairment, underscoring the need for differentiated and evidence-based prevention approaches.

Social environments play an important role in the normalization and interpretation of doping practices [7] Previous qualitative work has shown that IPED use is often embedded within specific risk environments shaped by peer networks, informal expertise and gym culture [19]. Within IPED-oriented peer groups and online communities, substance use is frequently framed as rational, disciplined, responsible, or even health-conscious, embedding risky behaviors into narratives of self-optimization and calculated decision-making [15, 36, 42]. Similar processes have also been discussed in research examining the relationship between supplement use and broader doping susceptibility in sport and exercise settings [5]. These framings may partly contribute to the limited effectiveness of deterrence-based approaches, particularly among experienced users who weigh perceived benefits against risks [14, 17, 35]. Consequently, prevention approaches increasingly emphasize harm reduction and risk communication rather than moral condemnation or prohibition [4, 18]. These dynamics intersect with broader public-health and regulatory discussions, particularly in contexts where IPED use occupies a grey zone between drug policy, informal harm-reduction practices, and commercial fitness culture. To ensure theoretical clarity, the present replication positions the Theory of Planned Behavior (TPB) as the central explanatory framework, complemented by selected constructs from the Health Belief Model (HBM) that capture health-related risk appraisal processes.

The Theory of Planned Behavior [2, 3] explains how attitudes, perceived norms, and perceived behavioral control are proposed to shape behavioral intentions, whereas the Health Belief Model [13, 38], emphasizes perceived susceptibility, perceived severity, and self-efficacy in relation to health-related decision-making. Previous TPB-based research has repeatedly demonstrated associations between attitudes, perceived norms and doping intentions in adolescent and sport populations [24]. Risk-related tolerance and protective orientations have been discussed in relation to both frameworks because they may influence how individuals interpret and respond to potential harms [6, 8, 9]. Within the present replication, TPB serves as the primary explanatory framework for intention-related orientations, whereas HBM-related constructs complement this perspective by capturing health-related risk appraisals and protective motivations. Together, these perspectives provide a complementary conceptual framework for examining doping-related decision-making in recreational fitness contexts.

While much prior research has relied on variable-centered approaches focusing on single predictors or prevalence estimation, such methods often overlook the motivational and cognitive heterogeneity within fitness populations. Person-centered approaches, in contrast, allow exploration of how motivational and cognitive dimensions may co-occur within individuals, thereby providing a more differentiated understanding of IPED-related orientations [10, 23, 28]. Building on this rationale, Paasch et al. [32] previously identified seven motivational and cognitive profiles among German fitness athletes based on TPB- and HBM-related constructs. However, the original study provided only preliminary information regarding cluster stability. The present replication therefore re-examines this typology using an independent sample and additional bootstrap and robustness analyses. Conceptually, these quantitative patterns are informed by qualitative evidence synthesized in a systematic review on amateur bodybuilding [30], which highlighted recurrent themes such as moral ambivalence, normalization of enhancement, and prevention fatigue. These motivational themes show descriptive similarities to classifications described by Ahmadi et al. [1] and may help contextualise profile-oriented prevention considerations.

Against this background, replication becomes particularly important. The growing replication and transparency movement within behavioral and health sciences has emphasized the importance of verifying psychological findings across independent samples and analytical procedures [26, 29]. In this context, replication extends methodological transparency into a field that remains empirically fragmented and where independent replications remain comparatively limited. Beyond reproducing earlier findings, replication enables refinement through additional robustness checks, reproducibility analyses, and improved cluster diagnostics [11]. In applied health psychology, replication may contribute to greater confidence in typological findings while reducing the likelihood of sample-specific artefacts [25, 40].

Building on these considerations, the present study aimed (1) to replicate and re-examine the previously established seven-profile typology of doping attitudes and prevention needs among German fitness athletes using selected TPB- and HBM-related constructs; (2) to examine cluster reproducibility and robustness using silhouette coefficients, bootstrap-based reproducibility analyses and algorithmic comparisons (Ward + k-means vs. k-medoids); and (3) to examine descriptive associations with lifetime use of anabolic–androgenic steroids (AAS), testosterone replacement therapy (TRT), and growth hormone (GH). To enrich interpretation, 52 open-ended responses were qualitatively analysed and used as illustrative examples of cluster-related motivational patterns. All measurement details, reliability coefficients, and clustering diagnostics are reported in the Supplementary Material to facilitate methodological transparency and reproducibility.

## Methods

### Participants and procedure

The sample comprised 624 recreational fitness athletes recruited from gyms and online platforms across Germany. Participants were 64.1% male, 34.5% female, and 1.4% diverse, with a mean age of 37.1 years (SD = 10.0; range = 18–65). On average, respondents reported 3.1 weekly training sessions (SD = 1.20) and 6.1 years of training experience (SD = 4.6). Training history was categorized as 0–2 years (19.9%), 3–5 years (38.1%), 6–10 years (27.7%), and >10 years (14.3%). These characteristics were broadly comparable to those reported in the original typology study [32].

Recruitment took place between August and September 2025 using a targeted offline–online strategy in cooperation with commercial fitness studios in Gelsenkirchen, Bochum, Essen, and Duisburg. Flyers containing QR codes directed participants to the online survey. Online recruitment occurred via regional Instagram and TikTok fitness groups as well as two NRW-based fitness forums. The invitation used neutral, non–doping-specific wording (e.g., “Survey on training, motivation, and health-related attitudes among recreational fitness athletes”). The survey was administered in German and primarily targeted German-speaking fitness populations. Because invitations were shared openly, the total number of individuals reached is unknown and no response rate can be calculated.

Eligibility criteria required participants to be at least 18 years old and to train in commercial gyms at least twice per week. Of the 624 completed surveys, 550 met predefined data-quality criteria and contained complete data on all clustering variables. No demographic differences emerged between included and excluded cases (all ps > 0.10).

### Data quality and exclusions

Of the initial 624 cases, exclusions were made based on predefined criteria: failed attention checks, excessive straight-lining, completion times below one-third of the sample median, and multivariate outliers identified via Mahalanobis distance (*p* < 0.001). The final analytical sample comprised 550 cases. Compared to the earlier dataset (N = 772), stricter criteria were applied to improve response consistency, data quality, and overall sample quality.

### Measures

Motivational and cognitive constructs were assessed using established scales derived from the Theory of Planned Behavior [2, 3] and the Health Belief Model [13, 38]. Intention and avoidance represented TPB-related components,risk perception reflected HBM-related components; and risk acceptance served as a descriptive indicator of harm tolerance and acceptance of potential adverse consequences [27, 33]. This construct was included because tolerance of potential harm has repeatedly been discussed in relation to enhancement-related decision-making in recreational fitness contexts.

No mechanistic pathways (e.g., mediation or moderation) between TPB- and HBM-related components were tested. All measures were identical to those used in the original typology. Items were rated on seven-point Likert scales (1 = strongly disagree, 7 = strongly agree). Internal consistency was satisfactory (α = 0.78–0.86). A confirmatory factor analysis indicated acceptable fit for a four-factor structure (CFI = 0.96, TLI = 0.95, RMSEA = 0.05, SRMR = 0.04).

Participants also reported weekly training frequency and training history. Lifetime use of anabolic–androgenic steroids (AAS), testosterone replacement therapy (TRT), and growth hormone (GH) was assessed dichotomously (0 = never, 1 = ever) and used as descriptive external correlates.

### Quantitative analyses

Analyses were conducted using SPSS 29. Following the original workflow, motivational and cognitive variables were z-standardized, hierarchical Ward clustering was applied, and the resulting centroids were used to initialize k-means clustering (k = 7, seed = 42). Descriptive cluster diagnostics were examined using the Calinski–Harabasz index [12], the Davies–Bouldin index [16], and the Gap statistic [41] to support interpretation of the retained solution (Calinski–Harabasz = 123.6,Davies–Bouldin = 1.26; Gap plateau = 6–8. Profile differentiation was explored using silhouette coefficients (overall = 0.22.

Importantly, cluster retention decisions in person-centered analyses are inherently multi-criterial and are rarely determined by a single numerical index alone. In the present study, silhouette coefficients, CH/DBI diagnostics, Gap statistic patterns, conceptual interpretability, and comparability with the previously reported typology were considered jointly when evaluating neighbouring clustering solutions.

Cluster reproducibility was explored using a predictive bootstrap procedure with B = 200 resamples [20]. For each bootstrap sample, a new seven-cluster k-means solution (seed = 42) was estimated and all original cases were reassigned to the nearest bootstrap centroids. Similarity between the original and bootstrap-based partitions was quantified using the Adjusted Rand Index (ARI). The resulting ARI distribution indicated moderate reproducibility of the seven-cluster solution, which was interpreted cautiously given the multidimensional and exploratory nature of the clustering procedure. Notably, neighbouring solutions (k = 5–8) also showed partially interpretable structures, indicating that the retained seven-profile solution should not be interpreted as uniquely optimal, but rather as one theoretically coherent and replication-compatible representation within a broader equivalence range. Moderate separation between profiles may also reflect the partially overlapping nature of motivational and cognitive orientations in real-world fitness populations rather than sharply distinct latent categories.

Descriptive differences between profiles on the clustering variables were examined using one-way ANOVAs. Because these variables directly informed the clustering procedure, findings were interpreted descriptively rather than as independent evidence of cluster validity. Associations with lifetime substance-use variables were examined descriptively using chi-square tests and Cramér’s V. Additional descriptive classification analyses were conducted using linear discriminant analysis (10-fold stratified cross-validation). Because predictors matched the clustering variables, these analyses were interpreted descriptively rather than as independent validation procedures.

### Qualitative illustration

A supplementary qualitative content analysis of 52 open-ended responses provided narrative elaboration of cluster-related motivational patterns. Coding followed a combined deductive (TPB/HBM-related) and inductive approach. Deductive codes included intention, avoidance, risk perception, risk acceptance, and social norms; inductive codes captured moral identity, rationalisation, and prevention needs. One trained coder performed all coding, and a subset of responses (15%) was revisited to enhance coding consistency and interpretive coherence. Given conceptual overlap with clustering variables and the single-coder design, this component was interpreted purely as illustrative rather than triangulating evidence.

### Transparency and reproducibility

All analyses used fixed seeds (seed = 42). Full SPSS syntax, preprocessing steps, CFA specifications, and cluster diagnostics are available upon reasonable request. Supplementary materials provide the codebook, validation plots, and detailed output.

## Results

The total sample comprised 624 participants (64.1% male, 34.5% female, 1.4% diverse; Mage = 37.1, SD = 10.0). Detailed sample characteristics are reported in Table 1.

**Table 1:** Sample characteristics of German fitness athletes (N = 624)

Variable	n (%)	M (SD)	Range
Age (years)	–	37.1 (10.0)	18–65
Gender: male	400 (64.1)	–	–
Gender: female	215 (34.5)	–	–
Gender: diverse	9 (1.4)	–	–
Weekly training frequency	–	3.1 (1.20)	0.0–6.4
Training years	–	6.1 (4.6)	0–20
0–2 years	124 (19.9)	–	–
3–5 years	238 (38.1)	–	–
6–10 years	173 (27.7)	–	–
10+ years	89 (14.3)	–	–

Percentages are based on the total sample of N = 624. Training years are reported as both mean values and categorical distributions. Due to missing values on clustering variables, the analytical sample used for cluster analyses was reduced to n = 550. All subsequent analyses are based on this cleaned analytical sample. No systematic differences in age, gender, or training history were observed between included and excluded cases (all ps > 0.10)

### Cluster analysis

A hierarchical Ward procedure followed by k-means clustering was conducted on the four TPB/HBM-related scales (intention, avoidance, risk acceptance, risk perception). A seven-cluster solution yielded a descriptively interpretable configuration that broadly aligned with the structure examined in the original typology study. The standardized centroids (z-scores) for all clustering variables are summarized in Supplementary Table S4, illustrating distinct motivational and cognitive patterns across the seven profiles.

**Fig. 1:**
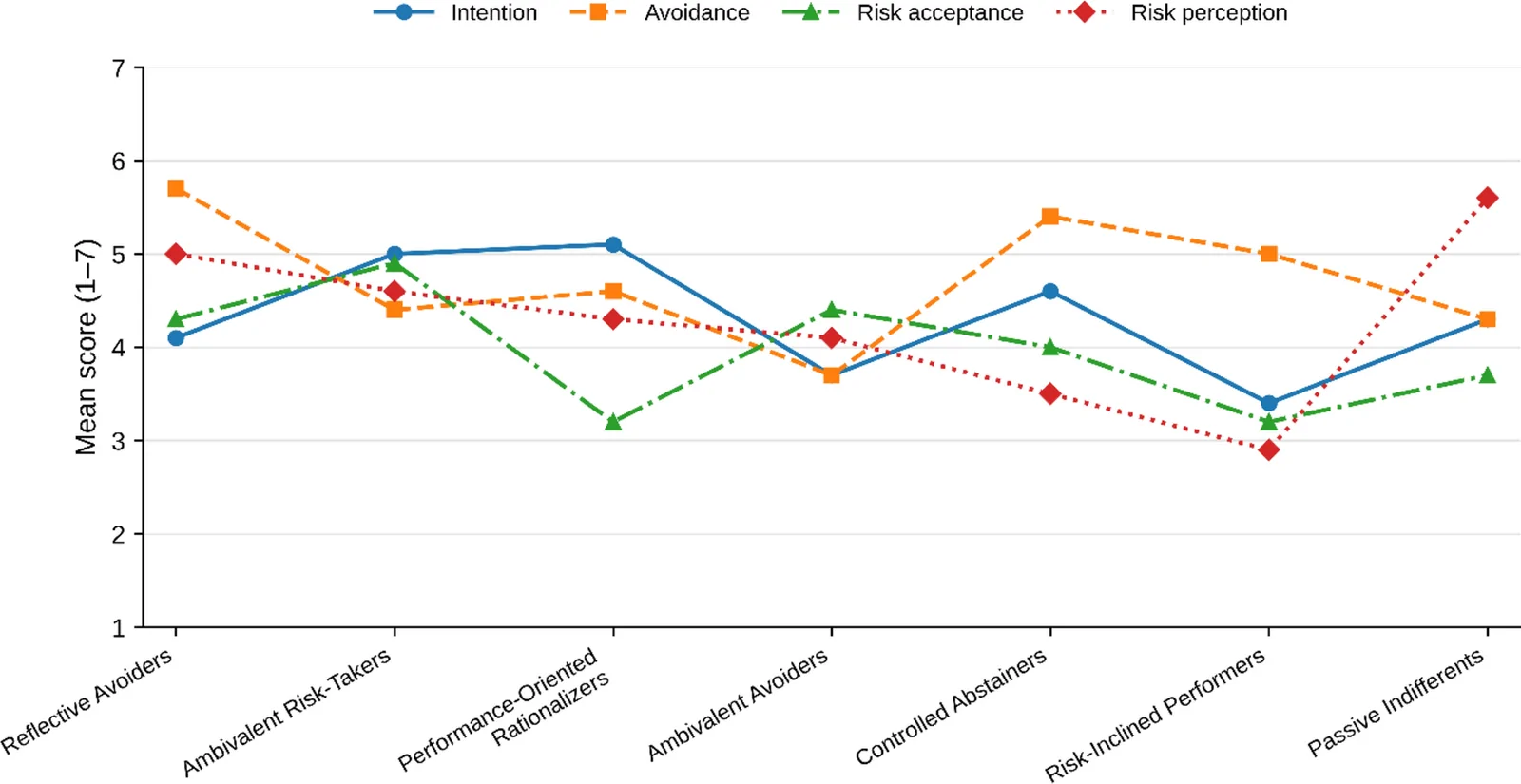
Mean scores for intention, avoidance, risk acceptance, and risk perception across the seven identified clusters. Note. The figure displays raw-scale means (1–7) corresponding to the descriptive values reported in Table 2. Cluster-specific demographic characteristics were broadly comparable across profiles (see Table 1)

To facilitate comparability with the original solution by Paasch et al. [32], Table 2 and Fig. 1 were structured analogously. The number and relative size of clusters were broadly comparable to the original distributio (approximately 8–16% per cluster), and the overall pattern of motivational and cognitive configurations showed broad descriptive similarities. For example, clusters corresponding to reflective or controlled abstainers again combined low intention and risk acceptance with higher avoidance, whereas performance-oriented profiles exhibited elevated intention scores, while risk-inclined profiles showed low intention but lower risk perception and greater tolerance for risk. Ambivalent types (Cluster 1 and 3) occupied intermediate positions between avoidance and risk acceptance. Taken together, these parallels suggest broad descriptive similarities to the main profile patterns observed in the original typology, while conclusions regarding structural stability should remain cautious given the moderate profile differentiation and exploratory nature of the analyses.

**Table 2:** Cluster means (M) and standard deviations (SD) for TPB/HBM constructs (intention, avoidance, risk acceptance, risk perception)

Cluster	N	Intention (M)	Avoidance (M)	Risk acceptance (M)	Risk perception (M)
Cluster 0 – reflective avoiders	60	4.1	5.7	4.3	5.0
Cluster 1 – ambivalent risk-takers	87	5.0	4.4	4.9	4.6
Cluster 2 – performance-oriented rationalizers	91	5.1	4.6	3.2	4.3
Cluster 3 – ambivalent avoiders	81	3.7	3.7	4.4	4.1
Cluster 4 – controlled abstainers	91	4.6	5.4	4.0	3.5
Cluster 5 – risk-inclined performers	70	3.4	5.0	3.2	2.9
Cluster 6 – passive indifferents	70	4.3	4.3	3.7	5.6

Cluster solutions based on standardized TPB/HBM scales (Intention, Avoidance, Risk Acceptance, Risk Perception). Values represent raw 1–7 means; clustering was performed on z-standardized inputs. n = cluster size

The overall silhouette coefficient was 0.22, with cluster-specific averages ranging from 0.15 to 0.27, indicating moderate profile differentiation typical for multidimensional survey data. Cluster sizes ranged from n = 60 to n = 91.

### Motivational and cognitive profile descriptions

Table 2 summarises the seven motivational and cognitive profiles based on standardized scores for intention, risk acceptance, risk perception, and avoidance. To avoid redundancy, we provide brief interpretive descriptions rather than repeating numerical values.

- *Cluster 0 – Reflective avoiders*
  Low intention and risk acceptance, high risk perception, strong avoidance. This group represents an abstinent, risk-aware profile pattern.
- *Cluster 1 – Ambivalent risk-takers*
  Moderate intentions, elevated risk acceptance, and mixed risk perceptions. Their profiles suggest cognitive ambivalence and decisional uncertainty.
- *Cluster 2 – Performance-oriented rationalizers*
  Higher intentions and comparatively low risk perception, combined with moderate avoidance. Responses within this profile were descriptively compatible with instrumental framings of IPED use.
- *Cluster 3 – Ambivalent avoiders*
  Lower intentions, low avoidance, and moderate risk acceptance. These individuals show situational uncertainty and variable avoidance tendencies.
- *Cluster 4 – Controlled abstainers*
  Moderately low intentions and risk acceptance, high avoidance, and lower risk perception. Abstinence appears grounded in discipline and self-regulation.
- *Cluster 5 – Risk-inclined performers*
  Relatively low intention and comparatively high avoidance, combined with the lowest risk perception across all clusters. These descriptive characteristics may indicate a tendency to downplay potential harms and a comparatively higher tolerance for risk, even though intention levels remain comparatively modest.
- *Cluster 6 – Passive indifferents*
  Moderate intentions with low topic engagement and high risk perception. Enhancement shows limited salience in their decision-making.

These profiles describe recurring motivational and cognitive configurations without duplicating the statistical information provided in Table 2 and Fig. 1.

Figure 1 displays the raw (1–7) mean scores for intention, avoidance, risk acceptance, and risk perception across the seven identified clusters. The figure complements Table 2 by visually illustrating the relative profile configurations across the included dimensions.

### Cluster stability (bootstrapping)

Cluster reproducibility was explored using a predictive nonparametric bootstrap procedure with B = 200 resamples, following recommendations by Hennig [20]. For each bootstrap sample of size N drawn with replacement, a new seven-cluster k-means solution (seed = 42) was estimated. All original cases were then reassigned to the nearest bootstrap centroids to obtain a full predicted partition for each resample. Similarity between the original and bootstrap-based partitions was quantified using the Adjusted Rand Index (ARI), which reflects the degree to which the original structure is recoverable across perturbed datasets.

Across the 200 resamples, the distribution of ARI values indicated moderate reproducibility of the seven-cluster solution, consistent with expectations for person-centered clustering of motivational and cognitive dimensions in heterogeneous survey samples. This pattern suggests moderate reproducibility of the overall profile structure, while individual cluster assignments should be interpreted cautiously. Because predictive bootstrapping does not estimate cluster-specific Jaccard indices, stability is evaluated at the global structure level rather than for each cluster individually.

### Cluster diagnostics and robustness checks

To further contextualise the retained seven-cluster solution, multiple descriptive clustering diagnostics were examined. Standardized centroids (z-scores) for all clustering variables are reported in Supplementary Table S4, illustrating the input space used in Ward pre-clustering and k-means optimisation (seed = 42).

The Calinski–Harabasz index (CH = 123.6) and the Davies–Bouldin index (DBI = 1.26) fell within ranges commonly reported in multidimensional clustering studies. Although the CH index peaked slightly at k = 5 (CH = 132.8), values for k = 6–7 remained similar, and a Gap statistic using five Monte Carlo reference datasets showed a plateau between k = 6 and k = 8. Within this equivalence range, the choice of k = 7 was guided primarily by replication logic and conceptual comparability with the original typology rather than by a single optimal numerical criterion.

To evaluate algorithmic robustness, the same data were re-analysed using Partitioning Around Medoids (k-medoids), which is less sensitive to outliers. Cluster assignments between k-means and k-medoids showed high descriptive similarity (Adjusted Rand Index = 0.89), suggesting that the observed groupings appear relatively stable across partitioning algorithms.

Visual inspection of standardized cluster means (see descriptive profiles in Table 2 and Supplementary Table S4) showed differentiated z-score patterns across intention, avoidance, risk acceptance, and risk perception. Taken together, these diagnostics indicate that the seven-cluster configuration represents a descriptively interpretable and theoretically comparable solution, while inferences about robustness and optimality should remain cautious.

### Comparison with original cluster typology

To enhance transparency, the replicated seven-cluster solution was systematically compared with the original typology published by Paasch et al. [32]. Supplementary Table S3 summarises both analyses side by side, highlighting methodological equivalence and additional diagnostics (CH, DBI, Gap statistic, exploratory bootstrapping). The comparison shows that the replicated solution showed descriptively comparable profile configurations and relative ordering of profiles observed previously, while also providing more detailed information on profile differentiation and algorithmic robustness. These correspondences suggest that central profile characteristics appeared descriptively comparable across samples, although conclusions about full structural replication should remain provisional.

Descriptive between-profile differences on the clustering variables are reported to characterise the relative positioning of the identified profiles. Because these variables were directly used in the clustering procedure, inferential interpretation of these differences should remain cautious and primarily descriptive.

These findings are descriptively compatible with theoretically plausible motivational and cognitive profile patterns derived from the Theory of Planned Behavior and the Health Belief Model. Moreover, the present patterns are compatible with prior qualitative syntheses of doping-related motivations [31], which highlighted the interplay of moral identity, rationalisation, and social context.

### Summary of cluster structure

Multiple descriptive clustering diagnostics indicated moderate profile differentiation typical for multidimensional survey data (overall silhouette = 0.22; cluster-specific values 0.15–0.27; see Table 3). Additional indices (CH, DBI, Gap statistic) showed comparable support for neighbouring cluster solutions, while cross-validated discriminant analyses indicated descriptively distinguishable profile configurations. Because these diagnostics partly rely on variables included in the clustering procedure itself, all findings are interpreted cautiously and descriptively rather than as confirmatory evidence of cluster validity.

**Table 3:** Cluster validity indices for the seven-cluster solution (silhouette by cluster; global CH and DBI reported in text)

Cluster	N	M (Silhouette)
0	60	0.15
1	87	0.27
2	91	0.22
3	81	0.26
4	91	0.18
5	70	0.21
6	70	0.23
Total	550	0.22 (overall)

Silhouette values represent average cohesion–separation indices per cluster

### Associations with lifetime substance use

To examine external associations, chi-square tests were conducted to assess links between cluster membership and lifetime use of AAS, TRT, and GH. Significant between-cluster differences emerged for AAS (χ^2^(6, n = 550) = 35.54, *p* < 0.001, V = 0.17) and TRT (χ^2^(6, n = 550) = 16.07, *p* = 0.013, V = 0.11), both representing small effects. GH use did not differ significantly across clusters (χ^2^(6, n = 550) = 11.53, *p* = 0.073, not significant). These findings indicate that while substance-use behaviours vary modestly across motivational and cognitive profiles, the observed effect sizes are small, suggesting that the profile structure primarily captures differences in belief structures rather than strong behavioural differentiation.

Associations with lifetime substance use are summarized in Table 4.

**Table 4:** Cross-tabulations of clusters by lifetime substance use (AAS, TRT, GH) with χ^2^ tests and Cramér’s V (n = 550)

Variable	χ^2^(df = 6)	*P*	Cramer’s V	Interpretation
AAS ever	35.54	< 0.001	0.17	Significant (small effect)
TRT ever	16.07	0.013	0.11	Significant (small effect)
GH ever	11.53	0.073	0.10	Not significant

χ^2^ = chi-square test; Cramér’s V = effect size (0.10 = small, 0.30 = medium, 0.50 = large). ns = not significant. AAS = anabolic–androgenic steroids; TRT = testosterone replacement therapy; GH = growth hormone

Taken together, these results suggest that cluster membership is associated with substance-use behaviour in small but statistically detectable ways. This pattern is consistent with the notion that motivational and cognitive orientations are more strongly differentiated than prevalence rates.

To enrich these quantitative findings, supplementary qualitative analyses were used to illustrate the motivational and interpretive nuances of each cluster.

### Qualitative results: illustrative analysis of open responses

To complement the quantitative replication, 52 open-ended responses were analysed to illustrate and contextualise how the seven clusters manifest in participants’ own language. This qualitative extension was not designed for hypothesis testing or independent validation, but to provide narrative examples consistent with the statistical profiles. The analysis followed a deductive–inductive approach: deductive categories were derived from TPB- and HBM-related constructs (e.g., intention, risk acceptance, risk perception, avoidance, social norms), while inductive categories captured recurring motives such as moral identity, rationalisation, and perceived prevention needs. The full set of coded responses is presented in Table S1, and category frequencies per cluster are displayed in Table S2.

- *Cluster 0 – Reflective avoiders*
  Responses frequently emphasised moral identity (57%), followed by risk perception (29%) and self-control (14%). Participants described abstinence as a moral principle and highlighted long-term health risks (e.g., “Long-term health is more important than short-term success.”). These patterns are descriptively compatible with a value-based abstinence orientation.
- *Cluster 1 – Ambivalent risk-takers*
  This group exhibited internal conflict, with codes for risk acceptance (33%) and risk perception (33%). Statements oscillated between curiosity and concern (“I swing between fear and fascination.”), suggesting experienced ambivalence and fluctuating evaluations of risk.
- *Cluster 2 – Performance-oriented rationalizers.*
  Participants often justified potential substance use through rationalisation and perceived training benefits (67%), while acknowledging some risk awareness (33%). Doping was sometimes framed as a controllable optimisation strategy (“Risks can be minimized through structure.”), illustrating instrumental reasoning patterns.
- *Cluster 3 – Ambivalent avoiders*
  Responses were frequently vague or inconsistent (≈ 60%), with some emphasis on social influence (≈ 20%). Participants described uncertainty and context-dependent reasoning (“My environment influences me more than I like.”), indicating situationally driven avoidance.
- *Cluster 4 – Controlled abstainers*
  Statements were dominated by moral identity and self-control (≈ 38% each), followed by planning and goal orientation (24%). Abstinence was framed as an expression of discipline and autonomy (“Clean training is a form of self-control for me.”), consistent with a self-regulatory perspective.
- *Cluster 5 – Risk-inclined performers*
  This group was characterised by relatively low intention with comparatively high avoidance but shows the lowest risk perception across all clusters. Qualitative responses were characterised by rationalisation (60%) and moderate risk perception (40%). Participants tended to downplay potential harms (e.g., “I feel in control; side effects are rare.”), reflecting a comparatively higher tolerance for risk despite comparatively modest intention levels.
- *Cluster 6 – Passive indifferents*
  About half of the responses reflected low intention and limited engagement (50%), with the remainder uncoded, suggesting a detached stance toward the topic (“I rarely inform myself, only when urgent.”). This pattern indicates low involvement and minimal deliberation regarding enhancement.

Overall, the qualitative material illustrates how motivational orientations ranging from moral conviction to calculated rationalisation and indifference are articulated in participants’ own words. These accounts add interpretive depth to the statistical profile configuration but do not constitute independent triangulation, as several deductive codes mirror the constructs used for clustering.

It is important to note that these qualitative patterns provide preliminary, hypothesis-generating indications rather than prescriptive or evidence-based prevention recommendations. Any cluster-specific considerations remain tentative and require empirical testing before informing concrete intervention design. The qualitative illustration therefore suggests possible directions for future prevention research, particularly regarding how moral identity, rationalisation, and ambivalence are expressed across motivational and cognitive profiles.

### Transition to discussion

Taken together, the quantitative and qualitative findings depict a heterogeneous motivational and cognitive landscape within recreational fitness doping. These combined findings provide an exploratory basis for the subsequent discussion, which situates the present results within the frameworks of the Theory of Planned Behavior and the Health Belief Model and considers implications for future research and prevention.

## Discussion

This study examined whether constructs from the Theory of Planned Behavior (TPB) and the Health Belief Model (HBM) can delineate distinct motivational and cognitive profiles among recreational fitness athletes and whether these profiles show parallels to previously documented patterns. Replicating typologies is essential for cumulative evidence building in a field where most studies rely on single samples and rarely test structural stability. Beyond this methodological contribution, the present findings also speak directly to current debates in drug policy and harm reduction. Rather than conceptualising IPED use as a homogeneous behaviour, the seven clusters reveal a differentiated motivational landscape in which abstinence, calculated risk-taking, ambivalence and disengagement coexist within the same gym culture. The observed between-cluster differences on TPB–HBM-related dimensions, combined with only moderate stability indices, suggest that doping-related orientations may reflect heterogeneous but partially overlapping motivational configurations, underscoring both the promise and the limits of typology-based segmentation for prevention and policy.

Although predictive bootstrapping provided additional insight into the robustness of the solution, the distribution of ARI values from the 200 resamples indicates only moderate structural reproducibility, which is typical for multidimensional clustering in heterogeneous survey samples. This pattern suggests that the seven-cluster configuration reflects a meaningful but probabilistic structure rather than sharply bounded categories. For drug-policy and harm-reduction contexts, this nuance matters: fluid and partially overlapping motivational-cognitive orientations cannot be addressed through singular prevention messages. Instead, the findings support flexible, context-sensitive approaches that account for shifts between ambivalence, risk tolerance and abstinence. Future replications should incorporate preregistered stability criteria and similarly robust or extended bootstrap procedures (e.g., B ≥ 200) to strengthen interpretive confidence and facilitate cumulative evidence building.

Several clusters showed close conceptual overlap with profiles described in recent IPED and harm-reduction research. Reflective Avoiders and Controlled Abstainers resembled groups driven by moral identity and health-protective norms, which tend to respond best to informational or normative prevention [8]. Ambivalent Risk-Takers and Performance-Oriented Rationalisers reflected patterns of instrumental reasoning, rationalisation and moral disengagement frequently reported in enhancement cultures [19]. Ambivalent Avoiders mirrored context-sensitive user groups whose decisions shift in response to situational cues and social influence. This interpretation is compatible with evidence suggesting that doping likelihood varies substantially across situational and self-referential contexts [37]. Risk-Inclined Performers showed comparatively elevated optimism-bias tendencies and comparatively elevated risk tolerance, traits linked to reduced responsiveness to deterrence-based interventions. Passive Indifferents resembled low-engagement groups documented in health-communication research, for whom IPED issues have low personal salience. Taken together, the clusters reinforce that IPED-related decisions do not follow a single linear risk continuum but instead reflect multiple, qualitatively distinct motivational pathways with direct implications for tailored harm-reduction strategies.

The qualitative accounts add narrative depth to these patterns. Participants described how they frame abstinence, ambivalence or calculated risk-taking in ways that align with earlier work demonstrating how users integrate risk knowledge into personalized harm-reduction logics [15]. These findings underline a central challenge for prevention: risk information alone is rarely sufficient if individuals interpret harms through identity-, performance- or community-based frames. For policy design, independent coding and structured sampling across user groups could deepen understanding of how narrative framings support or undermine formal harm-reduction messages.

### Theoretical interpretation

The clusters illustrate that motivational and cognitive orientations toward IPED use may reflect the interplay of planned decision-making and health-related risk appraisal processes rather than a single underlying mechanism. These orientations shape how individuals interpret harm-reduction messages, seek information, or develop safer-use routines. The present analysis does not test mechanistic relations between constructs but highlights distinct motivational constellations that offer multiple entry points for prevention. Risk acceptance may function less as a direct behavioural determinant and more as an indicator of uncertainty tolerance. Future confirmatory work using preregistered, model-based designs is needed to examine how these mechanisms translate into public-health outcomes.

### External associations and interpretive boundaries

Although cluster differences in lifetime AAS and TRT use were small, they remain meaningful from a harm-reduction standpoint. Dichotomous lifetime indicators capture only whether individuals have ever experimented and therefore obscure escalation patterns, dosage trajectories and polydrug practices that are central for policy [45]. The limited associations observed here suggest that the assessed motivational and cognitive orientations alone may not sufficiently capture behavioural variability, but may shape how individuals interpret risks, engage with prevention resources and adopt harm-reduction practices. For policy planning, typologies may help identify groups receptive to information-based prevention, those needing structural support, and those unlikely to respond to deterrence-based communication. More granular behavioural measures, including frequency, intensity, motives, and harm-reduction routines, are required to assess the practical relevance of motivational and cognitive profiles.

### Conceptual implications

The findings show that motivational and cognitive differentiation provides a valuable conceptual lens for understanding why individuals diverge in their engagement with IPED-related risks and prevention messages. Rather than functioning primarily as behavioural prediction models within the present design, the clusters highlight different ways athletes evaluate harm, negotiate social cues and regulate decision-making. This interpretation is also compatible with recent intervention research in recreational fitness settings indicating that theory-based and context-sensitive prevention approaches may improve responsiveness across heterogeneous user groups [32]. At the same time, these implications remain exploratory. The typology is not a diagnostic instrument and should not be used to classify individuals or prescribe specific intervention pathways. Instead, it provides a heuristic foundation for future hypothesis-driven work on how motivational and cognitive orientations shape responses to risk communication and harm-reduction efforts.

### Limitations

Several limitations should be acknowledged. First, the cross-sectional design precludes causal inference regarding motivational and cognitive constructs and doping-related behaviour. Longitudinal studies are needed to assess whether profiles remain stable or shift with changes in training environment, social context or risk perception. Second, reliance on self-report raises concerns about recall accuracy, social desirability and motivated reasoning, challenges well established in sensitive areas of drug research. Third, moderate cohesion–separation and only moderate bootstrap-based reproducibility suggest that the seven-cluster solution should be interpreted probabilistically. In addition, the identified profiles were not externally validated using independent behavioural or longitudinal outcome criteria and should therefore be interpreted as exploratory profile structures rather than fixed psychological categories. Fourth, the qualitative component was unevenly distributed, optional and coded by a single rater; it therefore adds illustrative depth but cannot serve as independent triangulation. Fifth, dichotomous lifetime-use measures compress behavioural variability, limiting the detection of graded risk trajectories. Sixth, cultural and regulatory specificity may restrict generalisability beyond German recreational fitness contexts. Finally, in the absence of preregistered replication criteria, no a priori thresholds for acceptable centroid similarity or stability were defined, limiting the strength of replication claims. These limitations underscore the need for preregistered, multi-method and longitudinal approaches incorporating richer behavioural indicators and independent qualitative validation to build a more cumulative evidence base for IPED-related motivational typologies.

### Future research

Future research should employ longitudinal and multi-wave designs to examine transitions between motivational and cognitive orientations and to explore how shifts in risk perception, social context or prevention messaging influence motivational patterns. Cross-cultural replications are needed to test the generalisability of the typology in settings with different regulatory climates. Independent qualitative studies using structured sampling and multiple coders could clarify how narrative framings shape harm appraisals and prevention engagement. Further work should also investigate how motivational and cognitive profiles moderate responses to different harm-reduction and policy strategies, including risk communication, safer-use guidance and environmental prevention strategies within gym settings [6]. Recent European mapping work further suggests substantial heterogeneity in anti-doping practices across fitness and gym settings [22]. Mixed-methods trials, ecological momentary assessments and digital self-monitoring tools may enhance contextual precision. Developing preregistered replication criteria, richer behavioural measures and open data-syntax repositories would further strengthen methodological transparency and facilitate cumulative evidence building.

## Conclusion

This study provides evidence that recreational fitness athletes differ systematically in their motivational and cognitive orientations toward doping. Integrating TPB- and HBM-related constructs enabled a descriptive mapping of seven qualitatively distinct profiles, offering insight into motivational diversity rather than strong behavioural prediction. While the typology should be interpreted cautiously and viewed as exploratory, it provides a structured foundation for future research on how attitudes, norms, and risk appraisals interact in shaping doping-related decision-making. Advancing this work will require longitudinal, preregistered, and multi-method designs that strengthen methodological rigour and support precision-oriented harm-reduction and policy strategies.

## Supplementary Information

Additional file 1.

## Data Availability

The datasets generated and analyzed during the current study are not publicly available due to privacy and ethical restrictions but are available from the corresponding author on reasonable request.

## Abbreviations

AAS: Anabolic-androgenic steroids
α: Cronbach’s Alpha
ANOVA: Analysis of variance
ARI: Adjusted rand index (cluster stability)
B: Number of bootstrap resamples
CH: Calinski–Harabasz index
CFA: Confirmatory factor analysis
CFI: Comparative fit index
DBI: Davies–Bouldin index
df: Degrees of freedom
EPPM: Extended parallel process model
GH: Growth hormone
HBM: Health belief model
IPEDs: Image- and performance-enhancing drugs
k: Number of clusters in k-means
LDA: Linear discriminant analysis
M: Mean
N / n: Sample size (total / subgroup)
PBC: Perceived behavioral control (TPB)
PEH: Performance enhancement & health (journal)
PES: Performance-enhancing substances
PWT: Prototype willingness theory
RMSEA: Root mean square error of approximation
SD: Standard deviation
SE: Standard error
SPSS: Statistical package for the social sciences
SRMR: Standardized root mean square residual
TLI: Tucker–Lewis index
TPB: Theory of planned behavior
TRT: Testosterone replacement therapy
WADA: World anti-doping agency
z: Z-standardized value
η^2^: Eta squared (effect size)

## Cluster labels used in this study

Cluster 0: Reflective avoiders
Cluster 1: Ambivalent risk-takers
Cluster 2: Performance-oriented rationalizers
Cluster 3: Ambivalent avoiders
Cluster 4: Controlled abstainers
Cluster 5: Risk-inclined performers
Cluster 6: Passive indifferents

## References

[1] Ahmadi A, Noetel M, Parker P, Ryan RM, Ntoumanis N, Reeve J, et al. A classification system for teachers’ motivational behaviors recommended in self-determination theory interventions. J Educ Psychol. 2023;115(8):1158–76. 10.1037/edu0000783.

[2] Ajzen I. The theory of planned behavior. Organ Behav Hum Decis Process. 1991;50(2):179–211. 10.1016/0749-5978(91)90020-T.

[3] Ajzen I. The theory of planned behavior: frequently asked questions. Hum Behav Emerg Technol. 2020;2(4):314–24. 10.1002/hbe2.195.

[4] Arno A, Thomas S. The efficacy of nudge theory strategies in influencing adult dietary behavior: a systematic review and meta-analysis. BMC Public Health. 2016;16:676. 10.1186/s12889-016-3272-x. PMC4967524 27475752

[5] Backhouse SH, Whitaker L, Petróczi A. Gateway to doping? Supplement use in the context of preferred competitive sport. Scand J Med Sci Sports. 2013;23(2):244–52. 10.1111/j.1600-0838.2011.01374.x. 22092778

[6] Bates G, Begley E, Tod D, Jones L, Leavey C, McVeigh J. A systematic review investigating the behaviour change strategies in interventions to prevent misuse of anabolic steroids. J Health Psychol. 2019;24(11):1595–612. 10.1177/1359105317737607. 29096544

[7] Bates G, Tod D, Leavey C, McVeigh J. An evidence-based socioecological framework to understand men’s use of anabolic androgenic steroids and inform interventions in this area. Drugs Educ Prev Policy. 2019;26(6):484–92. 10.1080/09687637.2018.1488947.

[8] Bates G, Ralphs R, Walker Bond V, Boardley I, Hope V, Van Hout M-C, et al. Systems mapping to understand complexity in the association between image and performance enhancing drugs (IPEDs) and harm. Int J Drug Policy. 2022;107:103801. 10.1016/j.drugpo.2022.103801. 35858502

[9] Blank C, Kopp M, Niedermeier M, Schnitzer M, Schobersberger W. Predictors of doping intentions, susceptibility, and behaviour of elite athletes: a meta-analytic review. Springerplus. 2016;5:1333. 10.1186/s40064-016-3000-0. PMC4980857 27563528

[10] Boardley ID, Grix J. Doping in bodybuilders: A qualitative investigation of facilitative psychosocial processes. Qualitat Res Sport, Exercise Health. 2014;6(3):422–39. 10.1080/2159676X.2013.796487.

[11] Brand R, Ekkekakis P. Affective–reflective theory of physical inactivity and exercise: foundations and preliminary evidence. Ger J Exerc Sport Res. 2018;48:48–58. 10.1007/s12662-017-0477-9.

[12] Caliński T, Harabasz J. A dendrite method for cluster analysis. Commun Stat. 1974;3(1):1–27. 10.1080/03610927408827101.

[13] Carpenter CJ. A meta-analysis of the effectiveness of health belief model variables in predicting behavior. Health Commun. 2010;25(8):661–9. 10.1080/10410236.2010.521906. 21153982

[14] Cox L, Piatkowski T. Enhancing autonomy through the ‘enhanced games.’ Perform Enhanc Health. 2024;12(2):100296. 10.1016/j.peh.2024.100296.

[15] Christiansen AV. *Bodybuilding and fitness doping in transition: Historical transformations and contemporary challenges* [PDF]. Syddansk Universitet. 2017. https://www.academia.edu/58473979/Bodybuilding_and_Fitness_Doping_in_Transition_Historical_Transformations_and_Contemporary_Challenges

[16] Davies DL, Bouldin DW. A cluster separation measure. IEEE Trans Pattern Anal Mach Intell. 1979;PAMI-1(2):224–7. 10.1109/TPAMI.1979.4766909. 21868852

[17] Forte T, Santinha G, Patrão M. Exploring the viability and acceptance of nudge in public policies for health promotion. Healthcare (Basel). 2024;12(4):476. 10.3390/healthcare12040476. PMC10887501 38391851

[18] Forberger S, Reisch LA, Kampfmann T, Zeeb H. Nudging to move: a scoping review of the use of choice architecture interventions to promote physical activity in the general population. Int J Behav Nutr Phys Act. 2019;16(1):77. 10.1186/s12966-019-0844-z. PMC6724306 31481090

[19] Hanley Santos G, Coomber R. The risk environment of anabolic-androgenic steroid users in the UK: examining motivations, practices and accounts of use. Int J Drug Policy. 2017;40:35–43. 10.1016/j.drugpo.2016.11.005. 27955960

[20] Hennig C. Cluster-wise assessment of cluster stability. Comput Stat Data Anal. 2007;52(1):258–71. 10.1016/j.csda.2006.11.025.

[21] Kanayama G, Brower KJ, Wood RI, Hudson JI, Pope HG Jr. Anabolic-androgenic steroid dependence: An emerging disorder. Addiction. 2009;104(12):1966–78. 10.1111/j.1360-0443.2009.02734.x. PMC2780436 19922565

[22] Lazuras L, Lauritzen F, Duiven E, Petrou M, Kozlovska L, Attuil J, et al. Anti-doping measures in gyms and fitness centres: a mapping of practices in the state parties of the Council of Europe’s Anti-Doping Convention. Int J Drug Policy. 2025;145:105025. 10.1016/j.drugpo.2025.105025. 41016249

[23] Lisha NE, Sussman S. Relationship of high school and college sports participation with alcohol, tobacco, and illicit drug use: a review. Addict Behav. 2010;35(5):399–407. 10.1016/j.addbeh.2009.12.032. PMC3134407 20100638

[24] Lucidi F, Grano C, Leone L, Lombardo C, Pesce C. Determinants of the intention to use doping substances: an empirical contribution in a sample of Italian adolescents. Int J Sport Psychol. 2004;35(2):133–48.

[25] Makel MC, Plucker JA. Facts are more important than novelty: replication in the education sciences. Educ Res. 2014;43(6):304–16. 10.3102/0013189X14545513.

[26] Nosek BA, Hardwicke TE, Moshontz H, Allard A, Corker KS, Dreber A, et al. Replicability, robustness, and reproducibility in psychological science. Annu Rev Psychol. 2022;73:719–48. 10.1146/annurev-psych-020821-114157. 34665669

[27] Ntoumanis N, Ng JYY, Barkoukis V, Backhouse SH, Petróczi A, Maguire R, et al. Personal and psychosocial predictors of doping use in physical activity settings: A meta-analysis. Sports Med. 2014;44(11):1603–24. 10.1007/s40279-014-0240-4. 25138312

[28] Ntoumanis N, Dølven S, Barkoukis V, Backhouse SH, Maguire R, Ring C, et al. Psychosocial predictors of doping intentions and use in sport and exercise: a systematic review and meta-analysis. Br J Sports Med. 2024;59(1):33–44. 10.1136/bjsports-2023-107910. PMC11503166 38925889

[29] Open Science Collaboration. Estimating the reproducibility of psychological science. Science. 2015;349(6251):716. 10.1126/science.aac4716. 26315443

[30] Paasch R, Mau G, Korol O. Perspectives, motivations and behaviors of amateur bodybuilders regarding doping and anti-doping: a systematic review. Perform Enhanc Health. 2025. 10.1016/j.peh.2025.100336.

[31] Paasch R, Mau G, Korol O. Doping attitudes and prevention needs in recreational sports: a behavioral cluster analysis based on the Theory of Planned Behavior and risk acceptance. Perform Enhanc Health. 2026;14(1):100392. 10.1016/j.peh.2025.100392.

[32] Paasch R, Mau G, Korol O. A multimodal, theory-based prevention program for IPED use in recreational fitness: evidence from a German cluster-randomized study. Perform Enhanc Health. 2026;14(2):100406. 10.1016/j.peh.2025.100406.

[33] Petróczi A, Aidman EV. Measuring explicit attitude toward doping: review of the psychometric properties of the Performance Enhancement Attitude Scale. Psychol Sport Exerc. 2009;10(3):390–6. 10.1016/j.psychsport.2008.11.001.

[34] Pope HG, Wood RI, Rogol A, Nyberg F, Bowers L, Bhasin S. Adverse health consequences of performance-enhancing drugs: an Endocrine Society scientific statement. Endocr Rev. 2014;35(3):341–75. 10.1210/er.2013-1058. PMC4026349 24423981

[35] Pöppel K. Efficient ways to combat doping in a sports education context!? A systematic review on doping prevention measures focusing on young age groups. Front Sports Act Living. 2021;3:673452. 10.3389/fspor.2021.673452. PMC8716629 34977566

[36] Raggatt M, Wright CJC, Carrotte E, Jenkinson R, Mulgrew K, Prichard I, et al. I aspire to look and feel healthy like the posts convey”: engagement with fitness inspiration on social media and perceptions of its influence on health and wellbeing. BMC Public Health. 2018;18:1002. 10.1186/s12889-018-5930-7. PMC6086030 30097034

[37] Ring C, Kavussanu M, Lucidi S, Hurst P. Effects of personal and situational factors on self-referenced doping likelihood. Psychol Sport Exerc. 2019;40:191–201. 10.1016/j.psychsport.2018.11.003.

[38] Rosenstock IM. The health belief model and preventive health behavior. Health Educ Monogr. 1974;2(4):354–86. 10.1177/109019817400200405.

[39] Sagoe D, Molde H, Andreassen CS, Torsheim T, Pallesen S. The global epidemiology of anabolic-androgenic steroid use: a meta-analysis and meta-regression analysis. Ann Epidemiol. 2014;24(5):383–98. 10.1016/j.annepidem.2014.01.009. 24582699

[40] Schmidt S. Shall we really do it again? The powerful concept of replication is neglected in the social sciences. Rev Gen Psychol. 2009;13(2):90–100. 10.1037/a0015108.

[41] Tavares ASR, Serpa S, Horta L, Rosado A. Psychosocial factors and performance enhancing substances in gym users: A systematic review. Revista de Psicología del Deporte. 2019;28(1):131–42.

[42] Tibshirani R, Walther G, Hastie T. Estimating the number of clusters in a dataset via the gap statistic. J Royal Stat Soc: Series B (Stat Methodol). 2001;63(2):411–23. 10.1111/1467-9868.00293.

[43] van de Ven K, Mulrooney KJD. Social suppliers: exploring the cultural contours of the performance and image enhancing drug (PIED) market among bodybuilders in the Netherlands and Belgium. Int J Drug Policy. 2017;50:48–55. 10.1016/j.drugpo.2016.07.009. 27574781

[44] World Medical Association. World Medical Association Declaration of Helsinki: Ethical principles for medical research involving human subjects. JAMA. 2013;310(20):2191–4. 10.1001/jama.2013.281053. 24141714

[45] Zahnow R, McVeigh J, Bates G, Hope V, Kean J, Campbell J, et al. Identifying a typology of men who use anabolic androgenic steroids (AAS). Int J Drug Policy. 2018;55:105–12. 10.1016/j.drugpo.2018.02.022. 29525360

