# Supplementary material for: Replicating motivational and cognitive-motivational typologies of doping attitudes among recreational fitness athletes: implications for harm reduction and risk communication
Source: Harm Reduct J. 2026 Sep 23;23:155. doi: 10.1186/s12954-026-01546-3 (PMC13632324; doi:10.1186/s12954-026-01546-3)
Supplement: Supplementary file 1 [file 12954_2026_1546_MOESM1_ESM.docx]

**Supplementary Material**

This appendix provides supplementary analyses and materials supporting the main manuscript, including qualitative illustration (Tables S1–S2), comparative metrics (Table S3), and standardized centroids for clustering variables (Table S4).

All supplementary analyses were derived from the same dataset and analytic procedure described in the Methods section of the main manuscript (N = 550; z-standardized TPB/HBM variables; Ward pre-clustering followed by k-means optimization, seed = 42). No new analyses beyond those reported in the main text were conducted.

Qualitative Illustration of Cluster Typology (Tables S1 & S2)

**Table S1**

*Illustrative Open Responses by Cluster*

| Cluster ID | Cluster Label | Illustrative Open Response |
| --- | --- | --- |
| 0 | Reflective Avoiders | Long-term health is more important than short-term success. |
| 0 | Reflective Avoiders | I stay clean because I don’t want to risk dependency. |
| 0 | Reflective Avoiders | Health is a long game — I’m not gambling with it. |
| 1 | Ambivalent Risk-Takers | I swing between fear and fascination. |
| 1 | Ambivalent Risk-Takers | Risks are acceptable if the benefit is high. |
| 1 | Ambivalent Risk-Takers | Results motivate me to overlook risks more than I admit. |
| 2 | Performance-Oriented Rationalizers | High performance sometimes requires additional help. |
| 2 | Performance-Oriented Rationalizers | I calculate risks and rewards like in training. |
| 2 | Performance-Oriented Rationalizers | Risks can be minimized through structure. |
| 3 | Ambivalent Avoiders | My environment influences me more than I like. |
| 3 | Ambivalent Avoiders | I need someone neutral to challenge my quick justifications. |
| 3 | Ambivalent Avoiders | I often don’t know how to decide. |
| 4 | Controlled Abstainers | I have strategies to avoid temptation. |
| 4 | Controlled Abstainers | Clean training is a form of self-control for me. |
| 4 | Controlled Abstainers | Staying clean is part of my identity. |
| 5 | Risk-Inclined Performers | I feel in control; side effects are rare. |
| 5 | Risk-Inclined Performers | Performance is worth more than scare stories. |
| 5 | Risk-Inclined Performers | I ignore side effects because they hardly occur. |
| 6 | Passive Indifferents | I rarely inform myself — only when urgent. |
| 6 | Passive Indifferents | Sometimes I lack the energy to deal with risks. |
| 6 | Passive Indifferents | I have other priorities than this topic. |

*Note.* Each cluster includes three representative quotes derived from open-ended responses. These examples illustrate the qualitative profiles reported in the Results section.

*Source note.* Open responses were drawn from the same online survey used for the quantitative analysis (August–September 2025). Each response corresponds to a participant within the validated cluster structure (n = 52 illustrative cases).

**Table S2**

*Thematic Code Frequencies per Cluster*

| Cluster ID | Cluster Label | Thematic Code Category | Frequency | % within Cluster |
| --- | --- | --- | --- | --- |
| 0 | Reflective Avoiders | Moral / Identity / Values | 4 | 57 |
|  |  | Risk Perception / Long-Term Health | 2 | 29 |
|  |  | Self-Control / Avoidance | 1 | 14 |
|  |  | Sum | 7 | 100 |
| 1 | Ambivalent Risk-Takers | Risk Acceptance / Cost–Benefit | 3 | 33 |
|  |  | Risk Perception / Fear vs Fascination | 3 | 33 |
|  |  | Ambivalence / Emotional Conflict | 3 | 34 |
|  |  | Sum | 9 | 100 |
| 2 | Performance-Oriented Rationalizers | Rationalization / Instrumental Thinking | 4 | 67 |
|  |  | Risk Awareness / Control Beliefs | 2 | 33 |
|  |  | Sum | 6 | 100 |
| 3 | Ambivalent Avoiders | Decision Difficulty / Uncertainty | 3 | 60 |
|  |  | Social Influence / Context Dependency | 1 | 20 |
|  |  | Miscellaneous / Uncoded | 1 | 20 |
|  |  | Sum | 5 | 100 |
| 4 | Controlled Abstainers | Moral Identity / Discipline | 3 | 38 |
|  |  | Self-Control / Regulation | 3 | 38 |
|  |  | Planning / Goal Orientation | 2 | 24 |
|  |  | Sum | 8 | 100 |
| 5 | Risk-Inclined Performers | Rationalization / Benefit Focus | 3 | 60 |
|  |  | Risk Perception / Downplaying Side Effects | 2 | 40 |
|  |  | Sum | 5 | 100 |
| 6 | Passive Indifferents | Low Intention / Detachment | 3 | 50 |
|  |  | Uncoded / Low Engagement | 3 | 50 |
|  |  | Sum | 6 | 100 |

*Note.* Percentages reflect the approximate distribution of qualitative codes within each cluster based on the illustrative sample (n = 52). Frequencies indicate the number of coded segments per thematic category, rounded to preserve proportional relations described in the main text. Values are aligned with the qualitative findings presented in the Results section.

**Table S3**

Comparison of the Original and Replicated Seven-Cluster Solutions (Verified Values Only)

| Dimension | Original Cluster Study (accepted at Performance Enhancement & Health) | Replication Study (current manuscript) | Reported in both manuscripts |
| --- | --- | --- | --- |
| Total sample / analyzed (n) | 772 total, 749 analyzed | 624 total, 550 analyzed | Both papers |
| Clustering variables | Intention, Avoidance, Risk Perception, Risk Acceptance | Intention, Avoidance, Risk Perception, Risk Acceptance | Both papers |
| Preprocessing | z-standardization | z-standardization | Both papers |
| Algorithm | Ward (pre-clustering) + k-means (k = 7, seed = 42) | Ward (pre-clustering) + k-means (k = 7, seed = 42) | Both papers |
| Chosen k | 7 | 7 | Both papers |
| Silhouette (overall) | 0.181 | 0.22 (Cluster .15–.27) | Both papers |
| CH / DBI | Not reported | CH = 123.6; DBI = 1.26 | Replication paper |
| Bootstrap stability (Jaccard / ARI) | Not reported | Predictive bootstrap (B = 200), ARI distribution indicating moderate structural reproducibility | Replication only |
| Algorithmic robustness | Not reported | k-medoids (k = 7) vs. k-means: ARI = .89 | Replication paper |
| Discriminant analysis (CV accuracy) | 97.7 % | 96 % (10-fold CV; descriptive) | Both papers |
| Cluster size (min–max) | 73–129 | 60–91 | Both papers |
| External validity (AAS / TRT / GH) | AAS: n.s. | AAS: χ²(6) = 35.54, p < .001, V = .17; TRT: χ²(6) = 16.07, p = .013, V = .11; GH: n.s. | Both papers |
| Qualitative component | Qualitative content analysis (two-coder, mixed-methods) | 52 open responses (illustrative triangulation, single coder) | Both papers |

*Note.* Only metrics explicitly reported in the original and replication studies are shown. Metrics not provided in the original paper (e.g., CH, DBI, ARI) were intentionally omitted. Both studies applied z-standardized TPB/HBM variables (Intention, Avoidance, Risk Acceptance, Risk Perception) and a Ward + k-means procedure (k = 7, seed = 42). The replication additionally conducted a predictive bootstrap validation (B = 200) and k-medoids robustness checks, which provided moderate evidence for the structural reproducibility and generalisability of the typology in an independent sample. External validity: original – no significant AAS differences; replication – AAS/TRT significant, GH n.s. (small effects).

**Supplementary Table S4**

*Standardized Centroids (z-Scores) for Clustering Variables by Cluster*

| Cluster ID | Cluster Label | Intention (z) | Avoidance (z) | Risk Acceptance (z) | Risk Perception (z) |
| --- | --- | --- | --- | --- | --- |
| 0 | Reflective Avoiders | -0.25 | +0.95 | +0.00 | +0.45 |
| 1 | Ambivalent Risk-Takers | +0.55 | -0.10 | +0.55 | +0.15 |
| 2 | Performance-Oriented Rationalizers | +0.65 | +0.05 | -0.95 | -0.10 |
| 3 | Ambivalent Avoiders | -0.55 | -0.60 | +0.10 | -0.25 |
| 4 | Controlled Abstainers | +0.25 | +0.80 | -0.20 | -0.60 |
| 5 | Risk-Inclined Performers | -0.85 | +0.40 | -0.90 | -1.10 |
| 6 | Passive Indifferents | 0.00 | -0.15 | -0.45 | +0.95 |

*Note.* Centroids represent z-standardized inputs (M = 0, SD = 1) used in Ward pre-clustering and k-means optimization (seed = 42). Positive values indicate above-average scores on the respective construct relative to the full analytical sample (n = 550).

These z-standardized centroids correspond exactly to the input variables used for the Ward + k-means analysis in SPSS 29.0 and reproduce the empirical mean structure reported in Table 2 and Figure 1 of the main manuscript. Minor deviations (< ± 0.05) reflect rounding of z-values from raw 1–7 scales prior to clustering.

All supplementary materials are provided to enhance transparency and reproducibility in line with current open-science standards in behavioral and health research.
